# Knowledge and attitude towards Ghana’s abortion law: A cross-sectional study among female undergraduate students

**DOI:** 10.1371/journal.pgph.0001719

**Published:** 2023-04-21

**Authors:** Shamsiyatu Abubakari, Vincent Uwumboriyhie Gmayinaam, Eric Osei

**Affiliations:** 1 Department of Epidemiology and Biostatistics, Fred N. Binka School of Public Health, University of Health and Allied Sciences, Hohoe, Ghana; 2 Department of Population and Behavioural Sciences, Fred N. Binka School of Public Health, University of Health and Allied Sciences, Hohoe, Ghana; University of Portsmouth, UNITED KINGDOM

## Abstract

Ghana amended its abortion law to permit abortion under certain circumstances due to the impacts of unsafe abortion. Even though the abortion law in Ghana is liberal, most women do not utilize the services. Studies have shown that lack of knowledge and attitude towards abortion laws are the barriers deterring women from using safe abortion services. This study, therefore, assessed the knowledge and attitudes of future female health professionals towards Ghana’s abortion law. This was an institutional-based cross-sectional study among 240 female students undertaking undergraduate courses at the Fred Newton Binka School of Public Health (FNBSPH), the University of Health and Allied Sciences (UHAS), Ghana. Knowledge was measured with 9 items using yes or no responses while Attitude was measured using a five-point Likert scale with 14 items. Factors associated with poor knowledge among the students were determined using logistic regression. All analyses were done using STATA version 16.0. Of the 240 participants, 24 (10%) reported ever being pregnant. Among these pregnancies, 20 (83.3%) ended in abortions, with 15 (75%) of them unsafely done. The majority (53.3%) of the students knew the conditions under which abortion is allowed in Ghana and most (61.7%) of them had positive attitudes towards the abortion legislation in the country. The year of study (aOR: 0.06; 95%CI: 0.01–0.23), residential status (aOR: 0.44; 95%CI: 0.20–0.74) and poor attitude towards the abortion law (Aor:0.46; 95% CI: 0.26–0.82) were associated with poor abortion law knowledge among the students. This study has demonstrated that knowledge and attitude towards Ghana’s abortion legislation among the students was fairly good. Students’ year of study, residential status and attitude towards the abortion law were also found associated with poor knowledge of Ghana’s abortion law. Increasing young women’s knowledge of the abortion law may lead to more favourable attitudes towards abortion, improving the utilization of safe and legal abortion services.

## Introduction

Women from all parts of the world, especially those in developing countries, lose their lives during pregnancy or childbirth [1] due to reasons that are preventable [2]. Globally, more than half a million maternal deaths occur annually [1,3], corresponding to about 830 deaths daily [4]. About 20% of maternal deaths in eighteen countries were linked to HIV [5], but complications from unsafe abortions are the second leading cause of maternal death in Ghana [6].

Unsafe abortion, as defined by the World Health Organization (WHO), is the termination of pregnancy either by persons lacking the necessary skills or in a setting that does not conform to minimal medical standards, or both [7]. Unsafe abortions represent about 13% of global maternal mortality, and it is very high in Africa [8,9].

Annually, over 4 million unsafe abortions are carried out in Africa, predominantly among poor, rural, and young women with no information on the availability of safe abortion services. Maternal deaths resulting from unsafe abortions are higher in Africa than in other developing countries [10]. In Ghana and other Sub-Saharan African countries, abortion is very prevalent, and several studies in the past have confirmed the extensive practice of unsafe abortions among different religious, ethnic and socioeconomic groups [10]. A study by Boah and colleagues showed that 64.1% of all abortions in Ghana are unsafe and induced [11].

Unsafe abortion is associated with complications that can result in death. Gebremedhin et al. posited that 20–50% of women from developing nations who engage in unsafe abortion get admitted to hospitals every year because of complications such as haemorrhage, sepsis, trauma to the cervix, vagina, uterus, and abdominal organs, peritonitis, and perforations [12]. Despite the medical consequences of unsafe abortion, it results in obviating socio-economic costs occurring at different social levels, such as the individual level, household and family level, healthcare system level, and the level for the development of communities and societies [13].

Deciding on the type of abortion procedure to choose is dependent on factors grouped at different levels. The individual-level factors influencing such decisions are the marital status of the victims and their economic independence and educational background. Partner and parental support are among the interpersonal determinants. Societal determining factors include social norms, religion, the stigma of premarital and extramarital sex on adolescents’ status, and autonomy in society. At the organizational level, the existence of sex education, health care systems, and abortion legislation affects a woman’s decision to abort a foetus [14].

Although abortion is legal in Ghana, Chandrasekaran and colleagues posited that most Ghanaian women were ignorant of the law and thought abortion was illegal [6]. Additionally, only 11% of women who heard about, or experienced abortion knew it was legal, according to the 2017 Ghana Maternal Health Survey report [15].

Most of the studies on abortion focused more on the determinants of unsafe abortion [16,17]. Moreover, there is little information about the knowledge and attitudes of the young women regarding abortion registration in Ghana. This study, therefore, sought to estimate the incidence of unsafe abortion and assess the knowledge and attitudes of undergraduate female students regarding Ghana’s abortion law in the Hohoe municipality, Ghana. The results of this study may inform policy makers about the extent to which young women know about the abortion law in the country and their attitudes towards it, which can help strategize information dissemination on how and where to obtain safe and legal abortion services.

## Materials and methods

### Study design and setting

An institutional-based cross-sectional study was conducted among students at the Fred Newton Binka School of Public Health (FNBSPH), University of Health and Allied Sciences (UHAS). The institution can be located in the Hohoe municipality, which is one of the 25 administrative districts of the Volta Region of Ghana. FNBSPH runs public health programmes including Disease Control, Health Information, Environmental Health, Public Health Nutrition, and Health Promotion at both the graduate and undergraduate levels. The school also runs top up undergraduate courses on sandwich bases to upgrade certificate and diploma holders. There were 1,125 undergraduate students in the school, among whom 549 were females [18].

### Study population

This study focused only on the female students pursuing undergraduate programmes at the school. Students who were apparently ill and required medical treatment during the data collection period and international students were excluded.

### Sample size determination

The Yamene’s formula for sample size determination was used to estimate the minimum sample size required for this study. The formula is given as n = N/1+N(e)^2^, where n is the required sample size; N is the size of the study population (549); and e is the margin of error (5%) at 95% confidence level. Based on this formula, the minimum sample size for the study was 231. However, 240 students took part in the study.

### Sampling method

For representativeness, stratified sampling was used to select participants. The list of female undergraduates was obtained and stratified according to years of study. Years of study were further stratified into the course of study. The participants selected were proportional to the population size of each stratum. Participants were selected using balloting without replacement from each stratum. Names of potential participants were written on pieces of paper and then folded and placed in a basket. The folded pieces were mixed to ensure uniformity. A neutral person was made to randomly select one folded paper at a time, and the basket was mixed again. The process was repeated until the required number of participants from each stratum was attained.

### Data collection tools and procedure

Data collection was done using a self-administered, structured online Google questionnaire. Class representatives served as key informants in identifying individuals within the samples. Contact details of the prospective participants were collected after consenting. Then the questionnaire was administered via their social media handle (WhatsApp). Participants were given a maximum of two (2) weeks to respond to the questionnaire.

The research instrument had four sections. The first section collected socio-demographic data, the second section included data on the prevalence and experiences of abortion; the third section included data on knowledge of abortion and abortion law, and the fourth section focused on students’ attitudes toward abortion and abortion law. Information on attitude level was collected using a five-point Likert scale. The choices ranged from strongly agree to strongly disagree and, this allows researchers to get a holistic view of people’s opinions. Knowledge was assessed with nine items using ‘yes’ or ‘no’ responses. The questionnaire was prepared in English and pretested on 10 students who were not part of the study population but had similar characteristics as the study population to check for consistency of variables and identification and correction of errors. The pre-testing enabled the researcher to know how clear the questions were and the estimated time to administer a questionnaire.

### Data analysis

Electronic data were downloaded into a single master database (Microsoft Excel 2016 spreadsheet) and Stata version 16.0 was used for analysis. The data were cleaned to remove errors and duplicates and checked for missing data. Descriptive statistics such as frequencies, proportions and percentages were performed on categorical variables, while means and standard deviations were used on quantitative variables.

In this study, an unsafe abortion was defined as a woman ending her pregnancy without medical help, such as by drinking herbal concoctions or using other home remedies, putting something in her vagina, or doing anything else to end the pregnancy by a nonmedical provider or in an environment that is not medically safe for that purpose [7].

Nine items were used to measure knowledge and a median score was computed in STATA version 16.0. Respondents who scored above or equal to the median were classified as having good knowledge and those who scored below the median were classified as having poor knowledge. Likewise, a mean score was calculated for attitude since the scores were normally distributed. Anyone who scored below the mean was classified as having an unfavourable attitude, while those who scored above or equal to the mean were considered as having a favourable attitude. Logistic regression was used to determine the factors associated with poor knowledge of Ghana’s abortion law among the students. First, a bivariate logistic regression was used to evaluate individual associations between predictor variables and the outcome variable (poor knowledge of abortion law). Secondly, a multivariate logistic regression model was fitted to adjust for the confounding effect of the predictors associated with knowledge of Ghana’s abortion law. Variables that showed a significant association at p<0.05 in the bivariate analysis were included in the final model. All tests were two-sided and a p-value less than 0.05 was considered statistically significant at the 95% confidence level.

### Ethical consideration

Participation in the study conformed to the required ethical guidelines regarding the use of human subjects. Ethical approval for the study was obtained from the University of Health and Allied Sciences Research Ethics Committee (UHAS-REC) (Reference Number: UHAS-REC A.9 [15] 20–21). Permission was obtained from the Head of School before the commencement of the study. Respondents were initially briefed and educated on the purpose and methods involved in the study and all their questions and concerns were addressed. The risks and benefits of the study were clearly explained, and the respondents had a right to refuse to participate or withdraw from the study at any time. Those who voluntarily agreed to participate then signed consent forms.

## Results

### Demographic characteristics of the respondents

Out of the 240 students studied, 143 (59.6%) were between the ages of 21–24, with a mean age of 21±1.9 years. Akans and Ewes were the majority (37.1%). Participants from households earning between 1000 and 2000 Ghana cedis per month were 29.6%, while 9.6% came from households earning less than 500 cedis per month. Nutrition students made up 33.8% of the study participants, with 75 (31.4%) being first-year students (Table 1).

**Table 1:** Demographic characteristics of respondents.

Variable	Frequency (N = 240)	Percentage (%)
**Age group (years)**		
18–20	81	33.8
21–24	143	59.6
25–29	16	6.6
**Marital status**		
Single	225	93.8
Married	3	1.2
Cohabitating	12	5.0
**Religion**		
Catholic	27	11.3
Other Christian	183	76.3
Islam	28	11.6
No religion	2	0.8
**Ethnicity**		
Ewe	89	37.1
Akan	89	37.1
Dagbani	10	4.1
Guan	11	4.6
Others	41	17.1
**Program of study**		
Disease control	80	33.3
Health promotion	42	17.5
Health information	37	15.4
Nutrition	81	33.8
**Year of study**		
Year 1	75	31.2
Year 2	71	29.6
Year 3	54	22.5
Year 4	40	16.7
**Residential status in school**		
Student’ hostel	73	30.4
Rented apartment	167	69.6
**Permanent residential status**		
Urban	195	81.3
Rural	45	18.7

#### Abortion experience among female undergraduates

More than half (53.3%) of the students had ever had sex as at the time of the study. Among these, 24 (18.8%) of them reported ever being pregnant. There were 20 abortions, 1 spontaneous and 19 induced. Among the induced abortions, 15(75%) were unsafe (Table 2).

**Table 2:** Abortion experiences among female undergraduates.

Variables	Frequency (N = 240)	Percentage (%)
**Ever had Sex**		
No	112	46.7
Yes	128	53.3
**Pregnancy experience (n = 128)**		
Ever been pregnant	24	18.8
Never been pregnant	104	81.3
**Ever had an abortion (n = 24)**		
Yes	20	83.3
No	4	16.7
**Had an abortion in the last 5 years (n = 20)**		
Yes	10	50.0
No	10	50.0
**Number of abortions done (n = 20)**		
1	15	75.0
2+	5	25.0
**Type of abortion (n = 20)**		
Induced	19	95.0
Spontaneous	1	5.0
**Gestational age at termination (n = 20)**		
1–3 weeks	7	35.0
4–5 weeks	5	25.0
6–8 weeks	8	40.0
**Place of Abortion (n = 20)**		
Home/self	13	65.0
Pharmacy	2	10.0
Private health facility	2	10.0
Public health facility	3	15.0
**Safety of last induced abortion (n = 19)**		
Safe	4	21.0
Unsafe	15	79.0

#### Knowledge of Ghana’s abortion law

**Fig 1:**
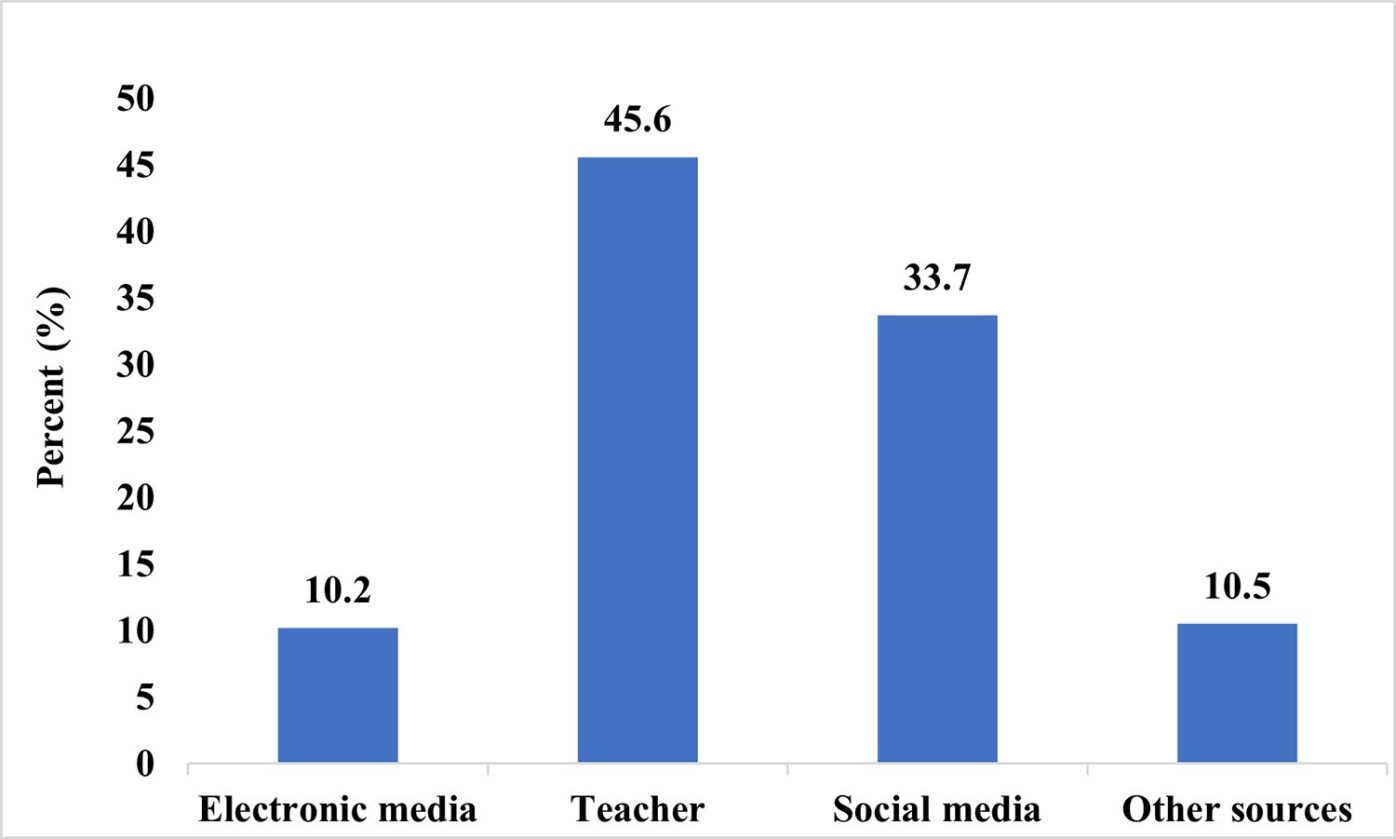
Sources of information on Ghana’s abortion law.

The median knowledge score was 6.0. Overall, the majority (53.3%) of the study participants had good knowledge of Ghana’s abortion law. School teachers (45.6%) were the most important source of information regarding Ghana’s abortion law (Fig 1). One hundred and eighty-seven (77.9%) of the respondents were aware of the legal status of abortion in Ghana. Among these, (75%) correctly stated that abortion is not legal in all circumstances in Ghana; Out of the total, 83% correctly stated that abortion in Ghana is legal in some circumstances; and 77% correctly stated that abortion is not illegal in Ghana in all circumstances (Table 3).

**Table 3:** Abortion law-related knowledge among respondents.

Variables	Yes	No	Don’t know
	n (%)	n (%)	n (%)
Aware of Ghana’s abortion law (n = 240)	187(77.9%)	53(22.1%)	0
Abortion is legal in all conditions in Ghana (n = 187)	19(10.2%)	141(75.4%)	27(14.4%)
Abortion is legal in some conditions in Ghana	155(82.9%)	15(8%)	17(9.1%)
Abortion is illegal in all conditions in Ghana	14(7.5%)	144(77%)	29(15.5%)
Ghana’s current abortion law permits a pregnant woman to terminate her pregnancy if raped	133(71.1%)	14(7.5%)	40(21.4%)
Current Ghana’s abortion law permits to terminate a pregnancy when a woman is endangered	144(77%)	10(5.3%)	33(17.7%)
Ghana’s abortion law permits to terminate a pregnancy when her faetus is endangered	143(76.5%)	8(4.3%)	36(19.3%)
Ghana’s abortion law permits to terminate a pregnancy when the woman is physically and psychologically unprepared	87(46.5%)	43(23%)	57(30.5%)
Ghana’s abortion law permits to terminate a pregnancy when the woman’s age is < 18 years	30(16%)	91(48.7%)	66(35.3%)
Ghana’s abortion law permits to terminate a pregnancy when the woman gets pregnant from her relatives	70(37.4%)	43(23%)	74(39.6%)

### Attitude towards Ghana’s abortion law

The average attitude score was 8.0. Overall, 61.7% of the students had a favourable attitude towards Ghana’s abortion legalisation. About 41% of study participants disagreed that a woman should be able to choose abortion if she is financially unable to care for her child; 39.1% disagreed that a woman should be able to terminate her pregnancy if she so desires. Safe abortion services in public health facilities, according to 51.7% of respondents, will increase promiscuity (Table 4).

**Table 4:** Ghana’s abortion law-related attitudes of respondents (n = 240).

Attitude items	Strongly agree	Agree	Neutral	Disagree	Strongly disagree
	n (%)	n (%)	n (%)	n (%)	n (%)
Safe and voluntary abortion should be legal and accessible.	80(33.3%)	70(29.2%)	46(19.2%)	31(12.9%)	13(5.4%)
Safe abortion is acceptable if a woman is financially unstable to cater for a child.	39(16.2%)	60(25%)	60(25%)	58(24.2%)	23(9.6%)
Safe abortion is acceptable to prevent a woman’s life or foetal abnormality.	120(50%)	81(33.8%)	24(10%)	11(4.5%)	4(1.7%)
A woman under 18 requesting a safe abortion service should be permitted to abort a pregnancy.	46(19.2%)	59(24.5%)	57(23.8%)	50(20.8%)	28(11.7%)
It is acceptable for a woman to choose safe abortion because of rape or incest.	102(42.5%)	78(32.5%)	28(11.7%)	23(9.6%)	9(3.7%)
A woman has the right to terminate her. pregnancy if she wishes	35(14.6%)	57(23.8%)	54(22.5%0	69(28.8%)	25(10.3%)
Abortion should not be allowed under any of the circumstances.	16(6.7%)	20(8.3%)	59(24.6%)	94(39.2%)	51(21.2%)
Making abortion services through the government health facilities may increase sexual immorality among people.	65(27.1%)	59(24.6%)	48(20%)	49(20.4%)	19(7.9%)
I will use the services if abortion is allowed legally.	58(24.2%)	71(29.6%)	62(25.8%)	33(13.8%)	16(6.6%)
Abortion should be available in the country on-demand only to those married.	11(4.6%)	21(8.8%)	58(24.2%)	108(45.2%)	42(17.2%)
Legal restrictions of the law drive people to unsafe abortions.	78(32.5%)	73(30.4%)	47(19.6%)	32(13.3%)	10(4.2%)
Abortion should be allowed out of health institution.	15(6.3%)	47(19.6%)	43(17.9%)	82(34.1%)	53(22.1%)
I will obey the current abortion law when I am pregnant and wish to terminate it.	51(21.3%)	68(28.3%)	70(29.2%)	33(13.8%)	18(7.4%)

#### Factors associated with poor knowledge of Ghana’s abortion law

A bivariate and multivariate analysis of factors influencing study participants’ poor knowledge is shown in Table 5. There was a statistically significant relationship between the year of study, caregiver residence, attitude; and poor knowledge with p-values of <0.001, 0.0014 and 0.001, respectively, in the bivariate analysis.

**Table 5:** Bivariate and multivariate analysis of factors associated with poor knowledge of Ghana’s abortion law.

Variables	Knowledge of Abortion law		cOR (95%CI)	P-value	aOR (95%CI)	P-value
	Good	Poor				
**Age group**				0.130		
** **18–20	39(30.5%)	42(33.0%)	**Ref**			
** **21–24	77(60.2%)	66(58.9%)	0.80(0.46,1.37)			
** **25–29	12(9.4%)	4(3.6%)	0.31(0.09,1.04)			
**Marital status**				0.483		
** **Married	1(0.8%)	2(1.8%)	**Ref**			
** **Unmarried	127(99.2%)	110(98.2%)	0.43(0.04,4.84)			
**Ethnicity **				0.634		
** **Akan	48(37.5%)	41(36.6%)	**Ref**			
** **Ewe	50(39.1%)	39(34.8%)	0.91(0.51,1.65)			
** **Others	30(23.4%)	32(28.6%)	1.25(0.65,2.39)			
**Program of study**				0.351		
** **Disease control	41(32.0%)	39(34.8%)	**Ref**			
** **Health information	24(18.7%)	13(11.6%)	0.57(0.26,1.27)			
** **Health promotion	24(18.7%)	18(16.1%)	0.79(0.37,1.67)			
Nutrition	39(30.5%)	42(37.5%)	1.13(0.61,2.10)			
**Year of study**				<0.001		
** **Year 1	28(21.9%)	47(41.9%)	**Ref**		**Ref**	
** **Year 2	32(25.0%)	39(34.8%)	0.73(0.38,1.41)		0.61(0.31,1.24)	0.177
** **Year 3	35(27.3%)	19(17.0%)	0.32(0.16,0.67)		0.33(0.15,0.70)	0.004
** **Year 4	33(25.8%)	7(6.3%)	0.13(0.05,0.32)		0.15(0.06,0.38)	<0.001
**Religion**				0.431		
** **Christian	111(86.7%)	99(88.4%)	**Ref**			
** **Islam	17(13.3%)	11(9.8%)	0.73(0.32, 1.62)			
**Monthly income**				0.979		
** **<500	12(9.4%)	11(9.8%)	**Ref**			
** **500–1000	38(29.7%)	32(28.6%)	0.92(0.36,2.36)			
** **1000+	78(60.9%)	69(61.6%)	0.97(0.40,2.33)			
**Type of Caregiver**				0.306		
** **Mother	17(13.3%)	24(21.4%)	**Ref**			
** **Father	8(6.3%)	8(8.0%)	0.80(0.26,2.48)			
** **Both parents	90(70.3%)	67(59.8%)	0.53(0.26,1.06)			
**Ever been pregnant**				0.216		
** **No	55(77.5%)	49(86.0%)	**Ref**			
** **Yes	16(22.5%)	8(14.0%)	0.56(0.22,1.43)			
**Permanent residential status**				0.001		
** **Rural	14(10.9%)	31(27.7%)	**Ref**		**Ref**	
** **Urban	128(89.1%)	81(72.3%)	0.32(0.16,0.64)		0.35(0.16,0.7)	0.007
**Attitude **				0.001		
** **Poor attitude	62 (48.4%)	78(69.6%)	**Ref**		**Ref**	
** **Good attitude	66(51.6%)	34(30.4%)	0.41(0.24,0.70)		0.39(0.22,0.70)	0.002

The multivariate analysis revealed that year of study, residential status, and attitude toward Ghana’s abortion law were the factors independently associated with poor knowledge of Ghana’s abortion law. Fourth-year students were less likely to have poor knowledge of Ghana’s abortion law compared to the first years (aOR = 0.15; 95% CI: (0.06–0.38); p<0.001). Respondents who grew up in urban areas were less likely than those from rural areas to have poor knowledge (aOR = 0.35; 95% CI: 0.16–0.70; p = 0.007). Respondents with a good attitude were less likely to have poor knowledge than those with a poor attitude (AOR = 0.39; 95% CI: 0.22–0.70; p = 0.002).

## Discussion

Despite the liberal nature of the abortion legislation in Ghana, unsafe abortion prevalence is still high. The prevalence of induced abortion in this study was 95% with 79% of all abortions being unsafe. This finding is not surprising since most cultural and religious groups in Ghana regard abortion as murder and ostracize those engaged in it. It also implies that most students resort to unsafe termination of pregnancy whenever they get pregnant since some even had more than one abortion [19]. The finding of the present study is a little higher than it was reported by Appiah- agyekum [20] among Legon students. This, therefore, justifies the Ghana Maternal Health Survey’s classification of students as the most vulnerable groups for practising induced abortions [16].

Similarly, Gelaye et al. reported in their study that induce abortion among female Wolaita Sodo University students was about 97% with nearly half of these abortions being unsafe [21].

A Ghanaian study among the entire population reported the prevalence of unsafe abortion to be 64.1% [11]. The difference in the various studies can be attributed to the use of different study populations. For instance, the previous Ghanaian study was conducted among women in the general population. Also, partly be explained by the type of study subjects. The burden of abortion in communities is disproportionately heavy on young people [22]. These findings imply that despite the attempt to reduce unsafe abortion and subsequent maternal mortality with Ghana’s abortion provisions in Act 29, criminal offences Act, 1960, sections 58 and 67, unsafe abortion still perturbs.

The high prevalence of unsafe abortion among university undergraduates is particularly troubling, as it may also imply that more of the students engage in unprotected and unsafe sex. This warns us to be on the mind of high risk of HIV/AIDS and other sexually transmitted diseases which has implications for the rising maternal death rate among women in Ghana and the poor reproductive health outcomes among females [23]. This, if persists may, in the long run, hinder Ghana’s progress towards achieving Sustainable development goal 3 target 1: reducing the global maternal mortality ratio to less than 70 per 100,000 live births. Similarly, abortion safety appears as a key contributor to the achieving of the aim of the safe motherhood initiative which was launched in Ghana by the Ghana Health Service in the year 1995 as a strategy to reduce maternal mortality and morbidity. However, the prevalence of unsafe abortion among these students is high. Also, the finding of this study draws the attention of health practitioners and health educators to the fact that unsafe abortion methods are the only acceptable method to students due to the stigma attached to pregnancy termination and students always prefer an undetectable abortion. Reproductive health practitioners should make efforts to destigmatize abortion to ensure the utilization of safe abortion services and reproductive health among students.

Ghana’s abortion law specifies the right procedures and places to conduct a safe abortion [24]. Safe abortion is one of the components of reproductive health that contributes to lowering maternal morbidity and mortality. While safe abortion is an important factor in reducing maternal mortality, its provision is governed by law. The awareness of this law has an impact on the use of safe abortion services [25]. In this study, about 5 in 10 of the respondents had good knowledge of Ghana’s abortion law. This finding is unsurprising, although it was reported in the 2017 Ghana Maternal Health Survey that only 11% of women of reproductive age knew abortion was legal in Ghana [26]. Our study participants are more knowledgeable than those of the Ghana Maternal Health Survey. Having good knowledge of abortion law implies that most of the students know where to get a safe abortion and in what circumstances they can access abortion care services when they have an unplanned pregnancy. This finding corroborates a study by Perera & Abeysena among undergraduates of state universities in the Western Province, Sri Lanka. The study revealed that most (53.3%) of the respondents had good knowledge of abortion legislation, while only 16.3% had poor knowledge of abortion law [27]. The good knowledge reported among the respondents of the present study can be attributed to reproductive health being added as a course to the curricula in the School of Public Health, UHAS. The similarity in both studies can be attributed to the fact that university students have access to more information, so they may know about abortion and its laws.

In contrast, previous studies in Ghana reported poor abortion law knowledge. This was reported in a study by Gbagbo, where only 33% of women knew abortion was legal in Ghana [28]. Similarly, a study by Reiger et al. among young women and men in Accra, Ghana, revealed that poor knowledge among respondents was high [6]. However, another similar study in Ethiopia reported that 79% of the respondents had poor knowledge of abortion law. The low level of abortion law can be attributed to the inability to raise awareness of the law and a lack of clarity in the law.

Good knowledge reported in this study also means that students are aware of the abortifacients used for terminating pregnancies and they mostly use them since they think these tablets have little to no risk. Health educators and other stakeholders, such as NGOs with an interest in reproductive health, should make it a point to inform these students that the tablet has its effects, although they are not as serious as those of other methods.

In this study, the majority (61.7%) of the respondents had a favourable attitude towards abortion legalization in Ghana. Most of the respondents agreed that abortion should be permitted to save the life of the woman as well as to prevent foetal abnormalities. The higher acceptance of the abortion legislation in this study may be attributed to the health-related academic discipline of the respondents. Similarly, an Ethiopian study reported that more than half (57%) of students in Ethiopia had a positive attitude towards abortion legalization [29]. This finding also corroborates with a Sri Lankan study, where the attitude towards abortion legalization was positive among the majority of the respondents [27]. Even though it was higher than the current study, 94.3% of respondents in a study done in Nepal were in favour of legalizing abortion [30]. Since a favourable attitude towards abortion legislation forms the basis for policies, health educators should direct their attention to providing counselling and education to students on the need to practice safe sex and also abstain. Policymakers should also strive for amendments to the law to include making abortion a right of the woman since sanctions drive most students to perform unsafe abortions.

The year of study predicted a poor knowledge level among the students. Fourth-year students were less likely to have poor knowledge of Ghana’s abortion law compared to those in their first year. This can be explained by the fact that females in the fourth year have an increased knowledge level compared to those in the first year since knowledge level generally increases with an increased level of education [29]. When compared to first-year students, fourth-year students may know more about abortion because they have heard more about it from student clubs on campus.

Urban dwellers were less likely to have poor knowledge of the legalization of abortion in Ghana compared to their rural counterparts. This can be explained by the fact that female undergraduates who live in cities with their caregivers have better access to information on reproductive health and may probe the legal status of abortion in the country; for that reason, they are more knowledgeable than those living in rural areas with their caregivers. This finding is in contrast with a study conducted among female students at Debre Markos University, Amhara Regional State, Northwest Ethiopia. The study revealed that female students whose families reside in rural areas were more knowledgeable than those in urban areas. The low knowledge level was attributed to social desirability bias or the failure of caregivers in urban areas to educate their girls on safe abortion. However, the difference between this study and the Ethiopian study may be attributed to differences in the socioeconomic status of both countries [31].

Further, attitude towards abortion legalization was found to be associated with knowledge level; females with a positive attitude were 40% less likely to have poor knowledge compared to those with an unfavourable attitude. This can be attributed to the fact that females with a favourable attitude may access information on the legal status of abortion in the country whenever they have an unplanned pregnancy.

This study is without limitations. Firstly, the data from this study may not represent all female undergraduates in Ghana because the study was limited to only one university. Secondly, the results of this study cannot be generalized to the general female population of Ghana, as the study population is relatively more educated and younger. Finally, due to the sensitive nature of abortion, social desirability bias and recall bias might have affected the results.

## Conclusion

The prevalence of unsafe abortion in this study was high, notwithstanding the fairly good knowledge and favourable attitudes regarding the abortion law of Ghana. Factors associated with poor knowledge were found to be the year of study, permanent residential status, and poor attitude towards the legislation. The need to intensify education on the abortion legislation and where women can get safe and legal abortion services cannot be overemphasised. Abortion services should be made available and accessible to the students.

## Supporting information

S1 Table Full multivariate analysis table. (DOCX)

S1 Data Original data used for the study. (XLSX)

S1 File Do file used to run the analysis. (DO)
