# Supplementary material for: Knowledge and attitude towards Ghana’s abortion law: A cross-sectional study among female undergraduate students
Source: PLOS Glob Public Health. 2023 Apr 21;3(4):e0001719. doi: 10.1371/journal.pgph.0001719 (PMC10121020; doi:10.1371/journal.pgph.0001719)
Supplement: Supplementary file 1 [file pgph.0001719.s001.docx]

```
.
. **************** Multivariate analysis ****************
. logistic KnowledgeVar i.Level i.Residence_caregiver i.AttitudeVar i.caregiver_type, baselevel

Logistic regression                                    Number of obs =    240
                                                       LR chi2(8)    =  54.31
                                                       Prob > chi2   = 0.0000
Log likelihood = -135.14542                            Pseudo R2     = 0.1673
```

KnowledgeVar	Odds ratio	Std. err.	z	P>|z|	[95% conf.	interval]
**Level**						
Level 100	1	(base)				
Level 200	.6042664	.2158165	-1.41	0.158	.3000668	1.216856
Level 300	.3846494	.1509218	-2.44	0.015	.1782732	.8299352
Level 400	.0734325	.0480877	-3.99	0.000	.0203458	.2650345
**Residence_caregiver**						
Rural	1	(base)				
Urban	.4362898	.1693906	-2.14	0.033	.2038421	.933805
**AttitudeVar**						
Poor attitude	1	(base)				
Good attitude	.3504503	.1091034	-3.37	0.001	.1903827	.6450976
**caregiver_type**						
Mother	1	(base)				
Father	.8136762	.5351831	-0.31	0.754	.2241748	2.95336
Both parents	.5022716	.2011611	-1.72	0.086	.2291027	1.101151
Other caregivers	.7510615	.4215885	-0.51	0.610	.2499632	2.256706
_cons	6.514687	3.454445	3.53	0.000	2.304304	18.41821
